# A functional extracellular transcriptome in animals? implications for biology, disease and medicine

**DOI:** 10.1186/s13059-015-0613-5

**Published:** 2015-02-26

**Authors:** Muneesh Tewari

**Affiliations:** Departments of Internal Medicine and Bioengineering, Biointerfaces Institute and Center for Computational Medicine and Bioinformatics, University of Michigan, 109 Zina Pitcher Place, 4029 BSRB, SPC 2200, Ann Arbor, MI 48109-2200 USA

## Abstract

Muneesh Tewari shares his views on possible functions and applications of RNA transport by extracellular vesicles in animals.

## Introduction

The past couple of years have seen a flurry of papers on RNAs that are transported by extracellular vesicles (ECVs) in animals. These RNAs have been implicated in intercellular communication in diverse biological contexts, from neuronal function to immunosuppressive effects of semen to cancer metastasis (see [1-3] for examples). Muneesh Tewari is an Associate Professor at the University of Michigan, where he studies extracellular RNAs in cancer and other diseases. He is currently involved in the NIH Extracellular RNA Communication Consortium, which focuses on comprehensively characterizing the human extracellular transcriptome as well as investigating the potential functions and applications of RNAs in ECVs. Here, he talks to *Genome Biology* about this exciting area of research and the challenges that need to be addressed.

## What has stimulated the recent interest in studying RNAs transported by extracellular vesicles?

The idea that there might be lateral transfer of nucleic acids between animal cells has been around for some time. As early as the 1970s there were reports that naked, purified mRNA could be taken up by mammalian cells and translated, though interest apparently waned over the next couple of decades and there was no satisfying mechanism proposed or any independent validation. Then, around 10 years ago, it became clear that in the nematode *Caenorhabditis elegans* short interfering RNAs are transported in between cells, and uptake of RNA from the environment was reported in both *C. elegans* and several insect species.

More recently, interest in mammalian RNA-based intercellular communication was stimulated by studies from Mariusz Ratajczak's lab in 2006 [4] and from Jan Lötvall's lab in 2007 [5] that not only suggested that RNA could be transferred between cells, but proposed as a mechanism that RNA is carried across in extracellular vesicles. Extracellular vesicles themselves have been known to exist for decades, and a subclass of these known as exosomes has been studied for some time in the context of being protein-presenting vehicles. But the finding of RNA being associated with ECVs was a new discovery. Ratajczak's team's studies of microvesicles from embryonic stem (ES) cells [4] suggested that the vesicles could transfer ES-cell-specific mRNAs into hematopoietic progenitor cells in a functional form. Lötvall's group [5] purified exosomes from a mast cell line and found that there were RNAs associated with these exosomes, including both non-coding (nc)RNAs - specifically micro (mi)RNAs - and mRNAs. In cell culture experiments they provided evidence that the mRNAs could be transferred to recipient cells in functional form, as evidenced by the production of corresponding proteins in the recipient cells. Soon after this, in 2008, Johan Skog and Xandra Breakefield [6] provided additional evidence. They looked at glioblastoma-associated extracellular vesicles from cells transfected with green fluorescent protein (GFP)-encoding plasmid DNA. They found that GFP mRNA was associated with these vesicles and, when added to recipient cells, the vesicles could deliver GFP mRNA to recipient cells in which it was then translated to protein.

So I think the recent surge in excitement has come from the idea that these vesicles may provide a mechanism for RNA-mediated intercellular communication. The basic idea is that RNA gets packaged into the vesicles, which are then released from the cell and travel (either locally or systemically like a hormone) to other cells, where the vesicles are taken up and functional RNA is delivered to the target cells in which it exerts its action. Once you have a possible mechanism there's a more directed path for investigation.

## Have technological advances been important?

As interest in extracellular vesicles has increased, the technology has also started to develop. The basic technologies were in place for studying small particles in other settings, far removed from extracellular vesicles in biology, and these technologies are now starting to be adapted for studying vesicles. An example of this is the use of nanoparticle tracking analysis technology to quantify and determine the size of exosomes, as well as the introduction of nanopore-based methods for similar purposes. The ability to globally characterize RNAs using profiling techniques such as RNA-seq has also contributed to progress in the field. Yet there are still many questions in the ECV field awaiting technology to answer them. I think we're going to see the development of more technologies for ECV analysis as the field matures.

## What do you think is the extent of this transport?

It's hard to say at this point. In experimental settings there are some very interesting data demonstrating such RNA transport, where purified preparations of extracellular vesicles are added to cells and taken up by the recipient cells along with the associated RNAs. But it's hard to say yet whether this is a physiological mechanism that is relevant in diverse biological contexts. Is it something that's widespread and that we were completely oblivious to before? Or is it going to turn out to be a niche mechanism that operates in very specific contexts or in experimental systems only?

## What kinds of ncRNAs are being found in these vesicles?

It's still very early in this field, but in the studies that have come out so far very diverse classes of RNAs are being reported. Not all studies find the same kinds of RNAs, though. So far sequences have been found that map to miRNAs, long non-coding (lnc)RNAs, other ncRNAs such as small nucleolar (sno)RNAs and tRNAs, as well as RNA reads mapping to mRNAs. So I think there's a high degree of diversity. That said, the studies are early.

It's worth noting than the NIH has initiated a Common Fund program in this area. Common Fund programs are intended to fund transdisciplinary projects in up and coming fields, for developing new paradigms as well as developing the new technologies needed to investigate them. The NIH has created a project called the Extracellular RNA Communication Program (http://commonfund.nih.gov/Exrna/index) and as part of this they are funding dozens of labs that will be taking up a range of questions and challenges around extracellular RNAs, including those found in vesicles. One of these challenges is to better characterize all extracellular RNAs: both RNAs that are associated with vesicles, and extracellular RNAs that are not associated with vesicles and that may be associated with proteins or other macromolecules [7,8]. My expectation is that in the coming years we will get a lot more data on the breadth of RNA species that are associated with various kinds of extracellular vesicles.

## What do we know about how certain ncRNAs get into vesicles for extracellular transport?

We really don't know much yet. There are some speculations at least for miRNAs, with respect to the miRNA effector machinery and where it localizes with respect to membranes inside cells, but really it's too early to say how they get loaded.

## How much evidence is there that the transported RNAs are functional at their destinations?

There is strong evidence that exosomes and other types of ECVs, when added to recipient cells, can mediate phenotypic effects. There's also evidence in experimental settings that some of these phenotypes can be mediated by ECV-associated RNAs [9]. But the jury is still out on the extent to which it is specifically RNAs in vesicles that mediate ECV-based modulation of target cells in natural physiologic settings.

## Might the RNA transport in ECVs have other roles?

One hypothesis that has been proposed is that one of the functions might be to rid the cell of certain RNAs. It's hard to say why the normal RNA degradation machinery in cells wouldn't be able to do this, but it would be interesting to look at whether there are some RNAs that are not degraded easily by the cell and whether these are preferentially enriched in vesicles. One could speculate that this might be especially the case for viral RNAs or other infectious RNAs that might escape the cellular RNA degradation machinery.

## What are the challenges in studying this phenomenon?

ECVs have been studied for some time but this intense focus on them is recent. As a result one basic challenge is nomenclature. The reality is that there are multiple types of ECVs and you can distinguish them by morphology and size using transmission electron microscopy. They also appear to have different mechanisms of biogenesis; a lot of this is now being teased out. The more we know about biogenesis the easier it will be to have clear and useful nomenclature and definitions for the different types of ECVs. In the meantime the International Society for Extracellular Vesicles (http://www.isevmeeting.org/) has come up with working definitions and nomenclature that they plan to refine on a regular basis.

Another challenge is analyzing the vesicles. There are technologies that can be used for this, as I mentioned, that in the past have been used for nanoparticle characterization, but they have their limitations. They are bulk technologies; for example, they can tell you how many vesicles there are in a population and their size distribution, but it is difficult to isolate and analyze a single vesicle in molecular detail. You also have to be careful because protein aggregates might sometimes show up as vesicles. Single vesicle analysis is very difficult, especially for smaller vesicles like classical exosomes that are smaller than the diffraction limit of light and can't be visualized by light microscopy. These smaller vesicles are the size of retroviral particles. We have lots of great technologies now to study single cells, such as flow cytometry, which can probably be used to study some of the larger vesicles, but single vesicle isolation and analysis is still a challenge.

Tracking and imaging vesicles *in vivo* is another challenge. We want to be able to see where they go and where they are taken up. Fluorescent labeling can be helpful for some of these studies but there is still more work to be done.

There is an additional, critical challenge for testing the hypothesis that RNA transport in ECVs is functional. Ideally, we would like to deplete ECVs of a specific RNA and determine how that affects the function of the ECVs for a given phenotype. We currently don't have any good ways of doing that. I think some of the CRISPR-CAS9 technologies will get us closer to that, and I expect we'll start seeing those studies in the near future.

## Can you speculate on how this transport might change our understanding of animal physiology?

If it turns out that a robust, ECV-based intercellular RNA transport mechanism is broadly operative in natural settings, it would change our understanding dramatically, because it suggests that the boundaries between cells are much more fluid than we previously thought. The absolute number of vesicles is very high: there can be on the order 10^12^ to 10^13^ of these particles per milliliter in the bloodstream. So although the vesicles are very small, numerically there are a lot of them around. If the hypothesis proves true, then it challenges the idea that organisms have compartmentalized cells. We know already that cells exchange information and molecules, but the boundaries might be much more fluid than we thought, between cells, tissues and organs.

For example, in cancer biology there is accumulating evidence that exosomes are secreted from a primary tumor and go to distant sites in the body, which they precondition for metastasis [10,11]. A key challenge is to determine how much the phenotype is due specifically to the RNA molecules associated with the vesicles versus other activities of exosomes - for example, through the proteins carried on the cell surface. In our own work we have found that when analyzed quantitatively, the absolute number of miRNA molecules associated on average with an exosome is tiny [12]. More work is needed to dissect the contribution of RNA associated with ECVs to the functional effects associated with ECVs.

## What are the implications for biomedicine?

Two main areas are being explored and both are the subject of large projects in the NIH Extracellular RNA Communication Program (http://commonfund.nih.gov/Exrna/index). One is therapeutic, the idea being that you can load ECVs with RNAs of therapeutic benefit and inject or otherwise deliver them for uptake by target cells. My own bias is that when we co-opt systems that have already evolved for a purpose we are interested in, it turns out to be successful more of the time than when we try to engineer systems. Thus, if ECVs are a natural, physiologic mechanism of RNA delivery, then I would expect that it could be very powerful therapeutically. There could also potentially be fewer safety concerns with this approach than with putting more artificial, synthetic materials into the body.

An important question is how much RNA you can load into ECVs. As mentioned above, we did a study of naturally occurring exosomes and tried to quantify how many RNA molecules on average are in each exosome, and we found that they were very scarce. However, this may not be true when you load exosomes experimentally.

A second area is diagnostics using RNA in vesicles. There are multiple companies now working on exosome-based diagnostics, trying to measure RNAs - not just ncRNAs but also mutated sequences in mRNAs in patients with cancer. Johan Skog and Xandra Breakefield in fact provided initial evidence for this approach in their paper in 2008 on glioblastoma-associated vesicles [6]. The idea is that cancer cells release vesicles into the circulation, so this is a way of sampling the tumor without having to do a biopsy. Vesicles would serve as replacement for a biopsy to look at gene expression profiles as well as specific mutant RNAs that are present. This could conceivably be used to look over time at the evolution of the tumor. There are some theoretical advantages over using circulating tumor DNA in that gene expression profiles measured from ECV-associated RNAs could provide more information about dynamic changes in tumor cell states than DNA, which tends to remain more static. But there are a lot of hurdles and unknowns. One challenge is isolating the vesicles that come from the tumor from the rest of the vesicles in the bloodstream - this can be challenging because there are a lot of background vesicles!
